# Optimum contribution selection for animal breeding and conservation: the R package optiSel

**DOI:** 10.1186/s12859-018-2450-5

**Published:** 2019-01-14

**Authors:** Robin Wellmann

**Affiliations:** Institute of Animal Science, University of Hohenheim, Garbenstraße, Stuttgart, Germany

**Keywords:** Optimum contribution selection, Animal breeding, Conservation, Segment-based kinship, Native kinship, Native contribution, Runs of homozygosity, optiSel

## Abstract

**Background:**

Selecting animals for breeding in the optimum way plays an essential role for the management of genetic resources and in selective breeding of livestock species. It requires to compute the optimum genetic contribution of each selection candidate to the next generation. Current software packages for optimum contribution selection (OCS) are not able to handle the main conflicting objectives of animal breeding programs simultaneously, which includes to increase genetic gain, to increase or to maintain genetic diversity, to recover the original genetic background of endangered breeds with historic introgression, and to maintain or increase genetic diversity at native alleles.

**Results:**

The free R package **optiSel** offers functions for estimating the above mentioned parameters from pedigree and marker data, and for solving OCS problems. One parameter can be optimized, whereas the remaining ones can be constrained. The results reveal the optimum numbers of offspring of all selection candidates, and can subsequently be used for mate allocation. Different solvers can be used. Solver slsqp was superior when the genetic diversity at native alleles was to be maximized, whereas solvers cccp and cccp2 were superior for all other OCS problems.

**Conclusion:**

Optimum contribution selection applied to local breeds requires special attention due to the conflicting objectives of their breeding programs. The free R package **optiSel** is an easy-to-use software taking these conflicting objectives into account.

**Electronic supplementary material:**

The online version of this article (10.1186/s12859-018-2450-5) contains supplementary material, which is available to authorized users.

## Background

The objectives of breeding programs for livestock breeds, companion animals, and zoo populations of endangered species may be quite different. In any case, however, selecting animals for breeding in the optimum way requires to compute the genetic contribution each selection candidate should have to the next generation.

For high-performance livestock breeds, the objective of a breeding program is to maximize genetic gain while at the same time a sufficient effective size of the breed should be maintained to avoid inbreeding depression or a depletion of the additive genetic variance. Maintenance of a sufficient effective size is achieved by restricting the rate of increase in mean kinship. Thus, the optimum contributions of the selection candidates are the solution of an optimization problem where the objective is to maximize the mean breeding value in the offspring while the increase in mean kinship in the population is constrained. This approach is the classical optimum contribution selection (OCS) proposed by [1].

High performance livestock breeds, however, have often been used for upgrading local breeds [2, 3]. This displacement crossing has often progressed to the point where the original genetic background of the local breed must be considered endangered. Hence, breeding programs for local breeds with historic introgression have the additional objective to recover the original genetic background of the breed. This means to reduce their genetic contribution from non-endangered breeds [4], to conserve the genetic diversity at native haplotype segments [5], and to maintain a sufficient genetic distance to non-endangered breeds [6].

In contrast, for many companion breeds (e.g. dog breeds), accurate breeding values for total merit are not available and historical genetic bottlenecks have depleted their gene pool. For these breeds, the main objective of the breeding program is to maintain or to increase genetic diversity by minimizing the mean kinship in the population. In this case, genetic introgression with other breeds may be not avoidable but should be restricted.

In summary, animal breeding programs can have different objectives simultanously, which are to increase genetic gain, to increase or to maintain genetic diversity, to recover the original genetic background of breeds with historic introgression, and to maintain or increase genetic diversity at native haplotype segments. Optimizing one of these criteria and restricting the others is called advanced OCS [7, 8].

Current software packages for OCS are not able to handle all conflicting objectives of animal breeding programs simultaneously and many of them may not find the global optimum. The implementation of classical OCS in the program **GenCont** uses Lagrangian multipliers [9], but is not guaranteed to find the optimal solution [10]. An alternative is the free software **EVA** [11] that uses an evolutionary algorithm for optimization. Methods using evolutionary algorithms are also described e.g., by [12] and are implemented in the commercial software **TGRM**. Some of these software packages provide flexible opportunities for mate allocation, but breeding programs that aim at recovering the native genetic background of a breed cannot be optimized with the software. An alternative is the use of general purpose software for optimization. Pong-Wong and Woolliams [10] demonstrated how OCS problems can be reformulated as semidefinite programming problems and used software **SDPA** [13] for optimization. Since the free software R is widely used by statisticians, of particular interest is general purpose software for optimization available as an R package. A variety of suitable packages exist. However, preparing animal data for use with general purpose software is a quite complex task, so it is rarely used by animal breeders or breeding organizations.

This paper introduces the free R package **optiSel** which provides a framework for solving advanced OCS problems with little R code. It also offers functions for estimating various parameters from pedigree and marker data. These are the kinships, kinships at native haplotype segments, and genetic contributions from native ancestors. The advanced OCS methods currently implemented include maximizing genetic gain, minimizing the average kinship, maximizing contributions from native ancestors, and minimizing the mean kinship at native haplotype segments, while criteria not included in the objective function can be used as constraints. This results in a table from which the optimum numbers of offspring of all selection candidates can be obtained, and which can subsequently be used for mate allocation to minimize the average inbreeding in the offspring.

The package enables to use a variety of free solvers for optimization and allows for easy switching between solvers by setting the parameter solver of function opticont() appropriately. Optimization problems can currently be solved by augmented lagrangian minimization as implemented in the R package **alabama** [14] (solver="alabama"), by semidefinite programming using the CSDP library introduced by [15] (solver="csdp"), by gradient-based optimization with sequential least-squares quadratic programming as implemented in function slsqp() [16] from package **nloptr** (solver="slsqp"), and by function cccp() from package **cccp** [17] for solving cone constrained convex programs (solver="cccp" or solver=~cccp2~).

The aims of this paper are to demonstrate how the free package optiSel can be used for the estimation of genetic parameters and for OCS. In addition, the suitability of the different solvers for solving a variety of OCS problems is compared.

## Implementation

The software package optiSel is implemented in R and C++. This section demonstrates the functionality of the package. This includes the estimation of genetic parameters and their use in OCS. Exact mathematical formulas for objective functions and constraints in OCS and their derivations can be found in (Wellmann R, Bennewitz J: Key genetic parameters for optimal population management, submitted).

The required packages optiSel and data.table can be downloaded from cran and then loaded as follows:


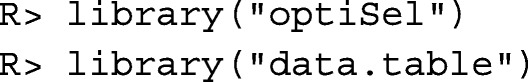


Package data.table is used because it provides a fast file reader. A simulated data set consisting of phenotypes, genotypes and pedigrees of simulated Angler cattle and a replication script can be found in the electronic appendix (Additional file 1). Estimation of genetic parameters and OCS are described below at the example of 1132 simulated genotyped individuals. Vector animals contains the IDs of these individuals. All estimated genetic parameters will be displayed for three related animals, which are an individual and its parents. These are the individuals included in vector I.


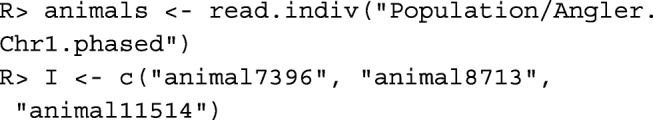


### Kinships

The kinship *f*_*IBD*_(*i*,*j*) of two individuals *i*,*j* is the probability that two alleles *X*_*i*_, and *Y*_*j*_, randomly chosen from both individuals from a single locus, are identical by descent (IBD). This means that they descend from a common ancestor. That is,

$$f_{IBD}(i, j) = P\left(X_{i} \stackrel{IBD}{=} Y_{j}\right). $$

Kinships can be estimated either from the pedigree or from marker data. In order to distinguish between segment-based estimates and pedigree-based estimates, we use for pedigree-based estimates the prefix or suffix PED, and for segment-based estimates SEG in this paper.

The **pedigree-based kinship** or **geneological coancestry**$\hat {f}_{PED}(i, j)$ between each pair of individuals *i*,*j* can be computed with function pedIBD(). The function allows to define a relationship matrix for the founders. By default, the founders are unrelated and not inbred. However, before a pedigree can be used, it needs to be prepared with function prePed(). This function sorts the pedigree, adds new lines for founders, and corrects some pedigree errors.


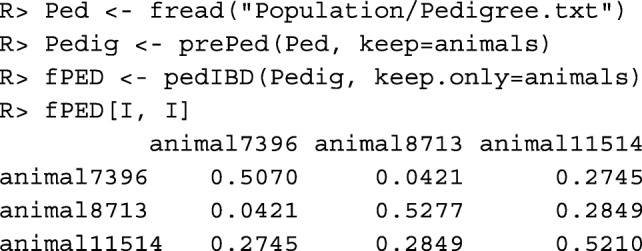


The additive relationship matrix A=2*fPED can also be computed with function makeA().

Pedigree-based evaluations require sufficiently complete pedigrees. Parameters quantifying the completeness of the pedigrees of all individuals can be obtained with function summary(). Of particular interest is the number of equivalent complete generations, which can be found in column equiGen. It is the sum of the proportions of known ancestors of an individual over all generations traced [18]. Below, data table phen, which contains the simulated breeding values in column EBV is loaded, and column equiGen is appended.


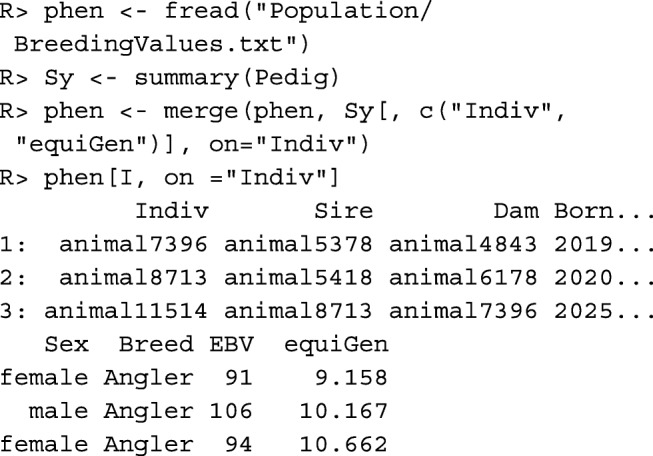


Pedigree-based estimates have the disadvantage that Mendelian sampling in all ancestors is considered to be random, so it cannot account for the alleles the ancestors actually inherited from their parents. In general, the usage of segment-based estimates is recommended in order to account for Mendelian sampling. The most useful marker-based kinship estimates are based on runs of homozygosity (ROH). A ROH with respect to two haplotypes is a segment consisting of consecutive base pairs which are identical in both haplotypes [19].

The **segment-based kinship**$\hat {f}_{SEG}(i, j)$ between individual *i* and *j* is the probability that two alleles, taken at random from both individuals from a single locus, belong to identical segments. The matrix containing the segment-based kinships of all individuals can be computed with function segIBD(). The number of cores to be used can be specified by argument cores, so different chromosomes can be processed in parallel.


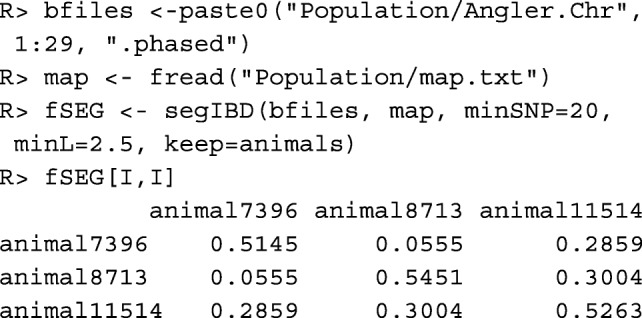


Important arguments of function segIBD() are minSNP and minL. A segment needs to have a minimum length for being taken into account. By default, the minimum number of markers to be included in a segment is minSNP=20 because considerably smaller sections of a haplotype may be identical by chance. The minimum length of a segment is by default minL=1.0 Mb. For the example data set we used minL=2.5 in accordance with [8]. Since short shared segments predominantly originate from early common ancestors, this value should be chosen depending on the age of the inbreeding that should be taken into account, but also dependent on the size of the marker panel [20].

### Native contribution

The native contribution *N*(*i*) of an individual *i* is the proportion of its genome which is native [8]. In other words, it is the genetic contribution it has from native ancestors, or the probability that an allele *X*_*i*_, randomly chosen from the individual, is native. That is,

$$N(i) = P\left(X_{i} \in \mathcal{A}_{N}\right), $$

where $\mathcal {A}_{N}$ is the set of alleles originating from native ancestors. It is usually defined with respect to a base population, i.e. a time *t*_0_ before which all registered individuals were considered native. Native contributions can be estimated either from pedigree or from marker data.

The **pedigree-based native contribution**$\hat {N}_{PED}(i)$ of individual *i* is the sum of the genetic contributions individual *i* has from native founders, whereby a founder is an individual with unknown parents. For estimating native contributions, the pedigree needs to be prepared differently than for estimating kinships. Below, arguments lastNative=1970 and thisBreed=~Angler~ ensure that the breed name of founders born after *t*_0_=1970 is shifted from ~Angler~ to "unknown". The native contributions and the contributions of other breeds to the genome of each individual are estimated with function pedBreedComp(). Thereafter, the column with native contributions is appended to data table phen and renamed as pedNC.


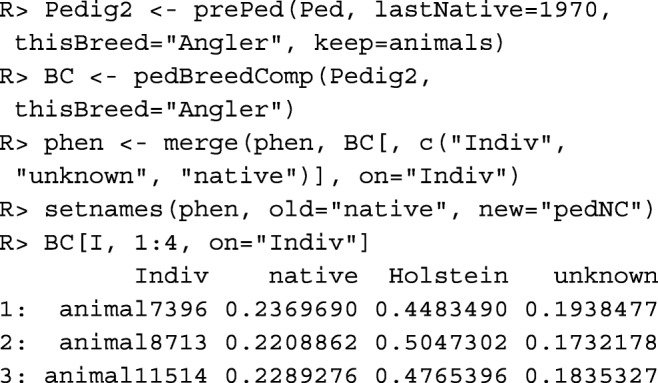


It can be seen that the selected individuals have a low native contribution, a high contribution from Holstein, and also a substantial contribution from individuals of unknown origin.

The **segment-based native contribution**$\hat {N}_{SEG}(i)$ of individual *i* is the proportion of its genome included in native haplotype sections. Thereby, an allele is considered native, if the segment containing the allele has low frequency in all breeds that might have been used for upgrading. That is, a marker *m* is native in a haplotype, if the frequency of the segment containing the marker is smaller than some threshold value ubFreq in all breeds that might have been used for upgrading the breed of interest. If a segment is substantially more frequent than (say) 0.01 in another non-endangered breed that was used for upgrading, then it does not need to be conserved and has likely been introgressed. Short segments predominantly arose from early introgression events, so segments are required to have a minimum length minL, which enables to neglect very old introgression.

Below, function haplofreq() is used to determine the most likely origin of each allele from each haplotype. The results are written to files in directory w.dir=~Population~, and a list with file names is returned. The first letters of the breed names are used in the files for labeling the origins of the markers, so care should be taken that these letters are different for the different breeds. Function segBreedComp() is used to compute the native contribution of each individual. Thereafter, the column with native contributions is appended to data table phen and renamed as segNC.


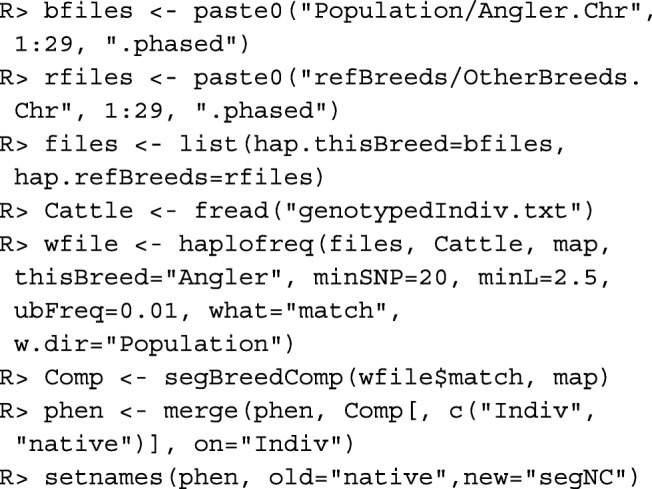


**Fig. 1:**
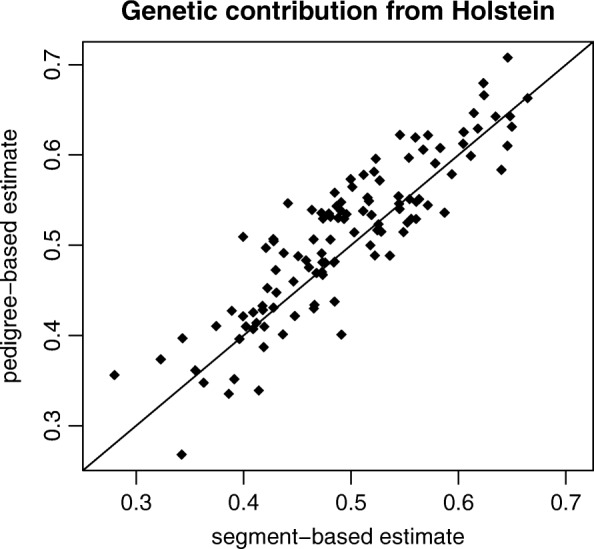
Joint Distribution. Pedigree-based estimates of the genetic contribution from Holstein cattle vs. segment-based estimates for simulated Angler cattle

The scatter plot in Fig. 1 shows the pedigree-based estimate of the genetic contribution from Holstein cattle vs. the segment-based estimate. Thereby, contributions from Holstein and Red Holstein are added and only individuals with real parents are included that have at least 6 equivalent complete generations in the pedigree. It can be seen that the segment-based contribution from Holstein is highly correlated with the pedigree-based estimate. Probably, both estimates are slightly biased downward. The pedigree-based estimate could be too low because of wrong and missing ancestors in the pedigree, whereas the marker-based estimate could be too low because some Holstein cattle with rare haplotypes are missing in the reference set.

### Native kinship

The native kinship *f*_*IBD*|*N*_(*i*,*j*) of two individuals *i*,*j* is the conditional probability that two alleles *X*_*i*_, and *Y*_*j*_, taken at random from both individuals from a single locus, are identical by descent (IBD), given that they are native. That is,

$$f_{IBD|N}(i, j) = P\left(X_{i} \stackrel{IBD}{=} Y_{j}\left|X_{i},Y_{j} \in \mathcal{A}_{N}\right.\right).$$

In other words, it is the kinship computed only from the alleles that are native in both individuals. Note that the native kinship depends neither on the way, the migrant ancestors were related with each other, nor on their genetic contribution to the population. Since the kinship is defined as a conditional probability, it can be computed by the ratio

$$\begin{array}{@{}rcl@{}} {f}_{IBD|N}(i, j) &=&\frac{{f}_{IBD\&N}(i, j)}{ {f}_{N}(i, j)}, \end{array} $$

where *f*_*IBD*&*N*_(*i*,*j*) is the probability that two alleles taken at random from both individuals are IBD and native, whereas *f*_*N*_(*i*,*j*) is the probability that both alleles are native. The numerator and the denominator, and thus the native kinships, can be estimated either from pedigree or from marker data.

The **pedigree-based native kinship**$\hat {f}_{PED|N}(i, j)$ between individuals *i*,*j* can be computed with function pedIBDatN(), whereby the native founders are assumed to be unrelated and non-inbred.


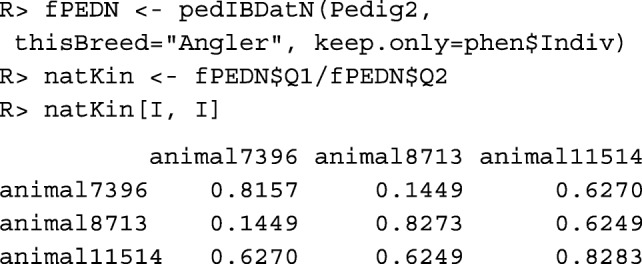


The native kinships of these individuals are rather high, which means that the sets of native ancestors in their pedigrees are considerably overlapping.

The **segment-based native kinship**$\hat {f}_{SEG|N}(i, j)$ between individuals *i*,*j* is the conditional probability that two alleles from the same locus taken at random from these individuals belong to identical segments, given that the alleles are native. It can be computed with function segIBDatN().


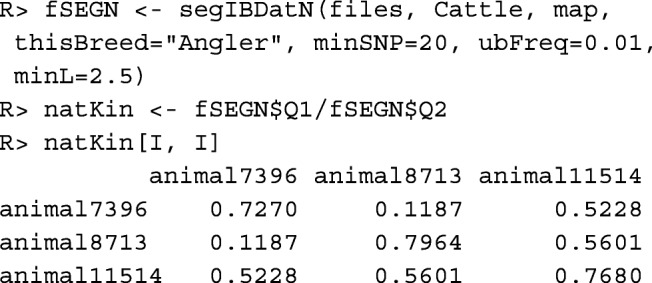


### Population means

The mean values of the genetic parameters in the population depend on the contributions the different age ×sex classes have to the population. The time interval covered by an age class needs to ensure that no individual can have offspring in the same age class. Typically, each age class spans one year.

Function agecont() estimates the contributions of the classes to the population. It assumes that the percentage of the population that is attributed to a particular class is proportional to the expected proportion of its offspring that is not yet born. Since these values are estimated from the past, this requires some continuity in the breeding program when this function is used for estimation. The total contributions of non-juvenile males and females to the population are assumed to be equal, whereby non-juvenile animals are all individuals that are not born in the current year. Note that the contributions are idealized and may not coincide with the proportions of living animals included in the classes. The contributions of the age classes are estimated from the ages of the parents at the time when their offspring was born. The offspring consists of the individuals indicated by argument use.


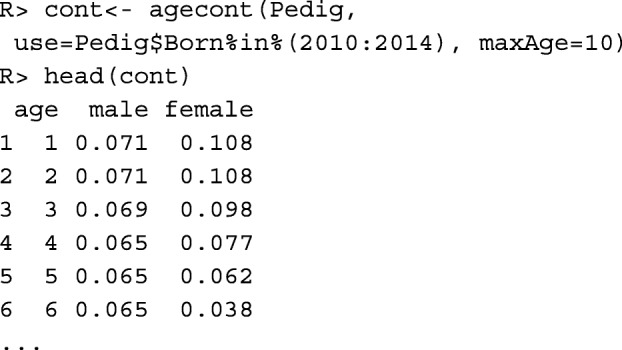


In this example, males have lower contributions to young age classes than females. This is because the males were predominantly progeny tested, so they were used for breeding at an older age. Hence, their contributions spread over a longer period of time.

Before we compute the population means, data frame phen should be completed by appending column isCandidate, which indicates the selection candidates for OCS. In this example, the selection candidates are the individuals that are at least one year old.


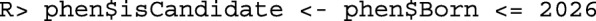


Function candes() computes the population means for all numeric columns in data table phen and for all kinships and native kinships that are supplied as additional arguments. Note that these additional arguments can have arbitrary names and they can be omitted if the respective kinship is not of interest. The population means depend on the contributions the different age ×sex classes have to the population as defined by argument cont. If argument cont is omitted, then discrete generations are assumed and the total contributions of males and females to the population are equal.


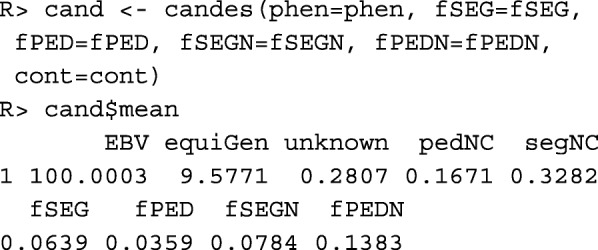


It can be seen that the average number of equivalent complete generations in the pedigree is rather high, even though the proportion of the genome with unknown origin is also moderately high. The results deviate from results of other studies for this breed [7, 8] because the data set used in this paper for demonstration purposes was not obtained from a random sample of the population. However, they demonstrate several interesting relationships between the parameters.

The pedigree-based native contribution of 0.1671 is probably underestimated because some of the founders with unknown origin may be native. The pedigree-based kinship is smaller than the segment-based kinship because pedigrees are incomplete. Native kinships are higher than the kinships because the diversity of native alleles is usually smaller than the total diversity of all alleles. The segment-based estimates of the native kinships are lower than the pedigree-based estimates. This has two reasons. First, the individuals have a substantial genetic contribution from founders with unknown origin. Alleles from these individuals do not contribute to the pedigree-based diversity of native alleles, even though some of them could have been native. This results in overestimating the pedigree-based native kinships. Second, crossing overs have shortened some haplotype segments, so that some segments can no longer be considered identical. This results in a slight underestimation of segment-based estimates.

### Constraint settings for kinships

Since the inbreeding coefficient of an individual is equal to the kinship of its parents, constraining the increase in mean kinship in the population enables breeders to avoid inbreeding. The rate of increase in mean kinship is measured by the variance effective size *N*_*e*_ of the population. The critical effective size, i.e. the size below which the fitness of the population steadily decreases, depends on the population and is usually assumed to be between 50 and 100 [21]. For most populations, maintenance of an effective size of *N*_*e*_≥100 should be envisaged. Hence, we define


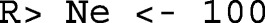


The effective size of the population is at least *N*_*e*_, if the rate of increase in mean kinship per generation is $\Delta f_{g} \leq \frac {1}{2N_{e}}$ [22]. In a population with overlapping generations and generation interval *L*, the rate of increase in mean kinship per year *Δf*_*y*_ is of interest for OCS, which should satisfy

$$\Delta f_{y} \leq \frac{1}{2N_{e}L}.$$

The generation interval can be approximated from the results of function agecont() as


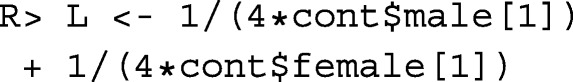


This enables to define upper bounds for the mean kinships in the population at the next evaluation time *t*+1 as


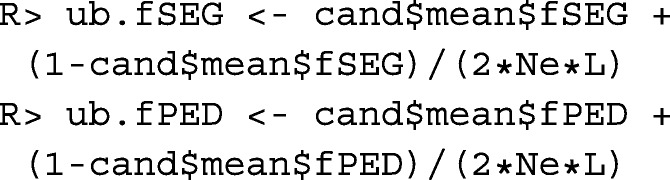


Of course, upper bounds need to be defined only for the parameters that should be constrained in OCS. The expected mean kinship in the population at time *t*+1 depends on the vector **c** containing the genetic contribution of each individual to the offspring, which is the parameter that will be optimized. The expected mean kinship can be computed by the quadratic function

$${f}_{{IBD}}(\mathbf{c}) = {\left(r_{0} \mathbf{c} + \mathbf{v}\right)}^{\top} \mathbf{f}_{{IBD}} {\left(r_{0} \mathbf{c} + \mathbf{v}\right)} + l_{{IBD}}(\mathbf{c}), $$

where *r*_0_ is the percentage of the population represented by the offspring, and component *v*_*i*_ of **v** is the percentage of the population represented by individual *i* itself. The small linear correction term *l*_*IBD*_(**c**) accounts, for example, for genetic drift (Wellmann R, Bennewitz J: Key genetic parameters for optimal population management, submitted). Estimates $\hat {f}_{PED}(\mathbf {c})$ and $\hat {f}_{SEG}(\mathbf {c})$ can be obtained by replacing **f**_*IBD*_ and *l*_*IBD*_(**c**) with their estimates obtained from pedigrees or marker data, respectively. Hence, constraining a kinship means to add a quadratic constraint of the form

$$\begin{array}{@{}rcl@{}} \hat{f}_{{PED}}(\mathbf{c}) &\leq& \text{ub.fPED}, \text{ or }\\ \hat{f}_{{SEG}}(\mathbf{c}) &\leq& \text{ub.fSEG} \end{array} $$

to the programming problem. Native kinships are of particular interest for populations with historic introgression if removal of the introgressed genetic material is envisaged in the future. Defining the upper bound for the mean kinship in accordance with the desired effective size ensures that enough genetic diversity will be maintained in the population after the introgressed genetic material has been removed. Hence, upper bounds are defined as


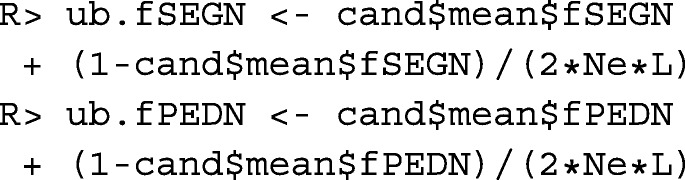


The expected mean native kinship in the population at time *t*+1 can be computed by the rational function

$$\begin{array}{@{}rcl@{}} {f}_{IBD|N}(\mathbf{c}) &=&\frac{{\left(r_{0} \mathbf{c} + \mathbf{v}\right)}^{\top} \mathbf{f}_{IBD\&N}{\left(r_{0} \mathbf{c} + \mathbf{v}\right)} + l_{IBD\&N}(\mathbf{c})}{ {\left(r_{0} \mathbf{c} + \mathbf{v}\right)}^{\top} \mathbf{f}_{N}{\left(r_{0} \mathbf{c} + \mathbf{v}\right)} + l_{N}(\mathbf{c})}, \end{array} $$

where *l*_*SEG*&*N*_(**c**) and *l*_*N*_(**c**) are the small linear correction terms defined in (Wellmann R, Bennewitz J: Key genetic parameters for optimal population management, submitted). Estimates $\hat {f}_{PED|N}(\mathbf {c})$ and $\hat {f}_{SEG|N}(\mathbf {c})$ are obtained by replacing the terms by their estimates obtained from pedigrees or marker data, respectively. Hence, constraining a native kinship means to add a rational constraint of the form

$$\begin{array}{@{}rcl@{}} \hat{f}_{PED|N}(\mathbf{c}) &\leq& \mathrm{ub.fPEDN}, \text{ or }\\ \hat{f}_{SEG|N}(\mathbf{c}) &\leq& \mathrm{ub.fSEGN} \end{array} $$

to the programming problem.

### Traditional OCS

The goal of OCS is finding the optimum contribution *c*_*i*_ each selection candidate *i* should have to the next birth cohort. It is the fraction of genes in the birth cohort that should originate from individual *i*. Since 50% of the genes originate from males and 50% originate from females, the proportion of individuals in the birth cohort having individual *i* as a parent should be 2*c*_*i*_.

Traditionally, OCS maximizes the mean breeding value in the population in the next year or generation, while the average kinship is required not to exceed a predefined threshold value. The usage of package optiSel is demonstrated below at the example of this optimization problem.

Since pedigree data is used, care must be taken that the completeness of the pedigrees is taken into account. Individuals with a low number of equivalent complete generations in their pedigree would otherwise be favored for breeding because they appear to be less related with the population. The constraints of the optimization problem are defined in a list:


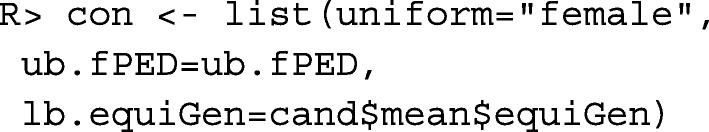


In this example, only the contributions of males are to be optimized, which is achieved by adding component uniform="female". That is, all females within a particular age cohort are assumed to have equal contributions to the offspring. This optimization problem is of interest if the contributions of the females cannot be centrally controlled.

Component ub.fPED=ub.fPED defines the upper bound for the mean kinship in the population to be equal to the value ub.fPED. This component has name ub.fPED because the kinship was named fPED in the call of function candes().

Component lb.equiGen=cand$mean$equiGen defines a lower bound for the average number of equivalent complete generations in the population. This constraint is only needed if incomplete pedigree data is used. The threshold value should be chosen such that individuals with incomplete pedigrees are not unduly favored for breeding. This component has name lb.equiGen because the column in data table cand$phen that contains the numbers of equivalent complete generations was named equiGen.

Optimization is carried out below with function opticont(). The first argument defines the objective of the optimization problem, which is to maximize the average breeding value in the population at time *t*+1. This is achieved with character string ~max.EBV~ because the column of data table cand$phen that contains the breeding values is named EBV.


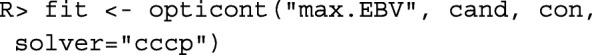


Argument solver defines the algorithm to be used for optimization. If numerical problems are encountered then it is advisable to use another solver or to adjust the tuning parameters of the solver, which can be supplied as additional arguments to function opticont(). Available solvers are

**cccp:** Function cccp() from R package **cccp** for solving cone constrained convex problems is called. Quadratic constraints are defined as second order cone constraints.

**cccp2:** Function cccp() from R package **cccp** is called, but quadratic constraints are defined by functions.

**alabama:** This solver calls function auglag() from R package **alabama** for optimizing smooth nonlinear objective functions with constraints.

**csdp:** This solver calls function csdp() from R package **Rcsdp** for solving semidefinite programming problems.

**slsqp:** Function slsqp() from package **nloptr** is called, which optimizes successive second-order approximations of the objective function with first-order approximations of the constraints.

The result of function opticont() is a list with several components. Data frame fit$info contains information on the success of the optimization. That is, component valid is TRUE, if all constraints are fulfilled by the optimized contributions, whereas component status describes the solution as reported by the solver.


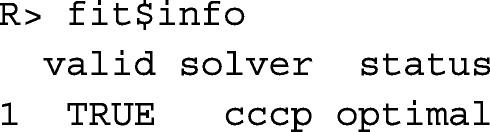


Data frame fit$mean contains the predicted mean values of heritable traits, kinships, and native kinships in the population at the next evaluation time *t*+1. For other variables, such as component equiGen, the weighted mean (*r*_0_**c**+**v**)^⊤^***X*** is shown, where ***X*** is the corresponding column vector from data frame cand$phen.


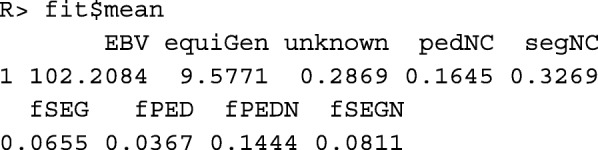


The optimized contributions of the breeding individuals can be found in column oc of data frame fit$parent:


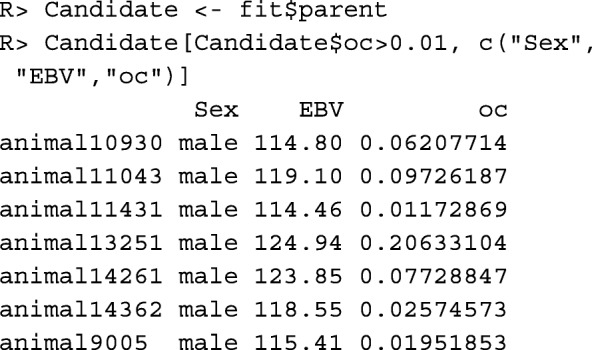


The example above optimizes only the contributions of males. For optimizing the contributions of both sexes, component uniform="female" needs to be removed from the list of constraints. Moreover, since the number of offspring a female can have is usually limited, upper limits need to be defined for the female contributions. More generally, upper and lower limits for the contributions of arbitrary individuals can be specified. If each birth cohort consists of *N*_0_=200 individuals and if a female can have at most 5 offspring per year, then the upper limit for the contributions of females needs to be $\frac {5}{2N_{0}}=0.0125$. The corresponding list of constraints can be created as follows:


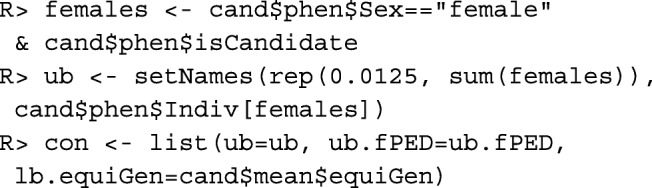


Computation of the optimum contributions with this list of constraints takes about 3 min. Their computation with constraint uniform="female" is in general much faster.

### Advanced OCS

This section provides an overview on the constraints and objective functions that can be handled by function opticont() and are of interest in many breeding programs. In general, all kinship and native kinships, and all numeric traits in data frame phen can be constrained. These parameters can also be optimized, but only one at a time. In animal breeding, the groups of males and females contribute equally to the offspring. This may, however, not be relevant in plant breeding. The constraint that males and females have equal contributions to the offspring is omitted, if column Sex in data frame phen contains only NA.

For most breeding programs, traditional OCS turned out to be not sufficient. This has several reasons. First, marker data enables to obtain more accurate estimates of kinships, native kinships and breeding values than pedigree data. In the examples below, we assume that marker data is available. However, if only pedigree data is available, then the examples can easily be adjusted by replacing terms SEG and seg with PED and ped. In particular, for maximizing breeding values while restricting the segment-based kinship, constraint ub.fPED=ub.fPED needs to be replaced with ub.fSEG=ub.fSEG:


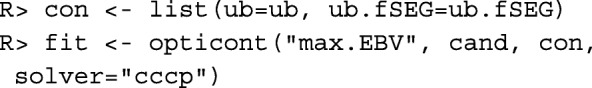


While the above setting is appropriate for most livestock breeds, many companion breeds and endangered breeds have different breeding objectives. Several companion breeds suffer from historic bottlenecks, which resulted in high inbreeding coefficients and inbreeding depression. For these breeds, the primary breeding goal is minimizing the average kinship in order to reduce inbreeding depression and the loss of genetic variation. This is achieved with the following call to function opticont():


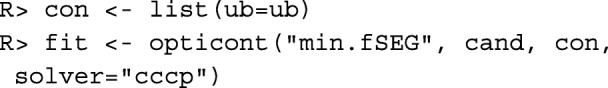


If breeding values are available, then they can be constrained in order to achieve genetic gain:


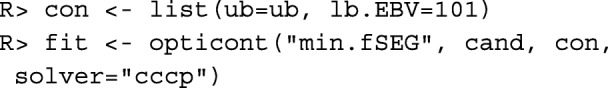


For some companion breeds, the erosion of genetic diversity has proceeded to a point that crossings with other breeds are not avoidable. However, the genetic contribution from other breeds should be restricted to the necessary minimum. Hence, a lower bound for the native contribution should be defined:


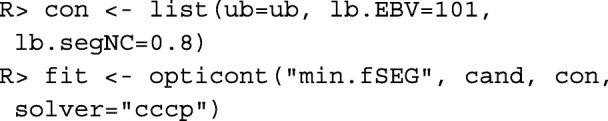


Note that the example above cannot be executed for the example data set because the optimization problem has no solution.

Some endangered livestock breeds, such as the breed used in the examples, have been continuously upgraded with high performance breeds in order to maintain economic competitiveness. Replacement of the original genetic background can have proceeded to the point that the original breed can be considered genetically extinct. For some of these breeds, de-extinction efforts are made with the aim to recover the original genetic background. Such breeding programs need to restrict the increase in native kinship in accordance with the desired effective size in order to ensure that enough genetic diversity persists in the breed after the foreign genetic material has been removed. Hence, the call to function opticont() would be


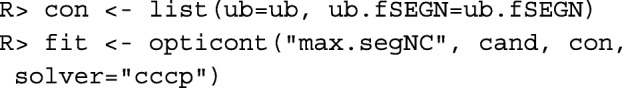


In general, recovering the native genetic background is not the only objective of the breeding program, but genetic gain should be achieved as well. In this case, breeding values for the native contribution should be estimated from marker data and included in the total merit index. Then, the total merit index would be maximized instead of the native contribution. It may be desirable to maintain specific introgressed QTL in the population, which could be achieved by giving them an appropriate weight in the total merit index.

### Mate allocation

After the optimum contributions of the selection candidates have been computed, males and females can be allocated for mating such that the mean inbreeding coefficient in the offspring is minimized. This can be done with function matings(). Since the kinship of the parents is equal to the inbreeding coefficient of the offspring, the objective is to minimize

$$\frac{1}{N_{0}} \sum\limits_{i\in\mathcal{M}}\sum\limits_{j\in\mathcal{F}} n_{ij} f_{ij}, $$

where *n*_*ij*_ is the number of offspring from the mating of individual *i* with individual *j*, $\mathcal {M}$ contains all male selection candidates, $\mathcal {F}$ contains all female selection candidates, *N*_0_ is the total number of offspring, and *f*_*ij*_ is either the segment-based kinship, or the pedigree-based kinship, or another user-supplied similarity measure for individuals *i* and *j*.

In any case, the genetic contribution of each parent must be equal to its optimum contribution. That is, for all males *i*, the following equation holds

$$\sum\limits_{j\in\mathcal{F}} n_{ij} = n_{i}, $$

and for all females *j*, we have

$$\sum\limits_{i\in\mathcal{M}} n_{ij} =n_{j}. $$

where *n*_*i*_≈2*c*_*i*_*N*_0_ is the number of offspring of individual *i*.

The maximum number of offspring per mating can be constrained to be ub.nOff at most. In this case, for all males *i*, and for all females *j* the following inequality holds

$$n_{ij} \leq \mathrm{ub.nOff}. $$

Without this constraint, some superior animals may always be mated to the same inferior individual, so all their offspring may not be good enough for breeding.

Moreover, for each herd, the proportion of offspring sired by the same male can be constrained to be at most *α*. This increases genetic connectedness between herds, so it enables to estimate more accurate breeding values. Take $\mathcal {F}_{h}$ to be the set of females from herd *h*. For all herds *h* and all males *i* we have

$$\sum\limits_{j\in\mathcal{F}_{h}} n_{ij} \leq \alpha \> N_{h}. $$

where *N*_*h*_ is the number of individuals in the birth cohort that will be born in herd *h*. Mate allocation is demonstrated at the example of OCS with segment-based kinship matrix. Recall that the optimization problem can be solved with


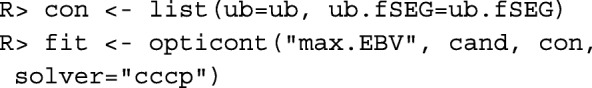


Function noffspring() is used below to compute the desired number of offspring per selection candidate by assuming that the birth cohort covers 200 individuals. The result of function matings(), which is used for mate allocation, is a data frame with columns Sire, Dam, and n. Column n contains the desired number of offspring from matings between the respective sire and dam. Note that this is the number of offspring that should be used as selection candidates in the next generation. The total number of offspring from the matings may be larger.


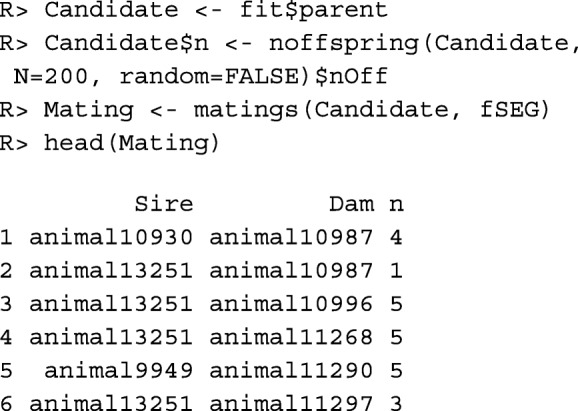


The average inbreeding coefficient of the offspring is


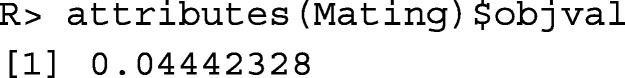


## Results

### Comparison of solvers

The ability of different solvers to find optimum solutions for different OCS problems was compared at the example of a data set containing genotypes, breeding values, and migrant contributions of 11000 simulated Angler cattle. These simulated individuals were generated from genotypes of 131 Angler bulls and 137 Angler cows during 2 generations of selection. Male selection candidates were sampled at random from the population that consisted of all 11000 individuals. Females were assumed to have equal contributions within each age class. Breeding values were simulated as described in [8]. Segment-based kinships, native kinships, and native contributions were estimated from haplotypes consisting of 23448 SNPs.

The following OCS-scenarios for populations with overlapping generations were considered:

**max.EBV:** This is traditional OCS with segment-based kinship matrix. The mean breeding value in population was maximized, while the mean kinship was constrained such that *N*_*e*_≥100.

**max.segNC:** This OCS approach is suitable for breeding programs whose main objective is to recover the native genetic background. The mean native contribution in the population was maximized, while the mean native kinship was constrained such that *N*_*e*_≥100.

**min.fSEG:** This objective function is suitable for breeds suffering from inbreeding depression. The mean kinship was minimized, while the mean native contribution was constrained, and the mean breeding value was constrained not to decrease.

**min.fSEGN:** This OCS approach may be suitable for breeding programs that aim at maximizing the genetic diversity at native alleles and at recovering the native genetic background. The mean kinship at native alleles was minimized, while the mean native contribution was constrained to increase by at least 2.5% per year.

**Fig. 2:**
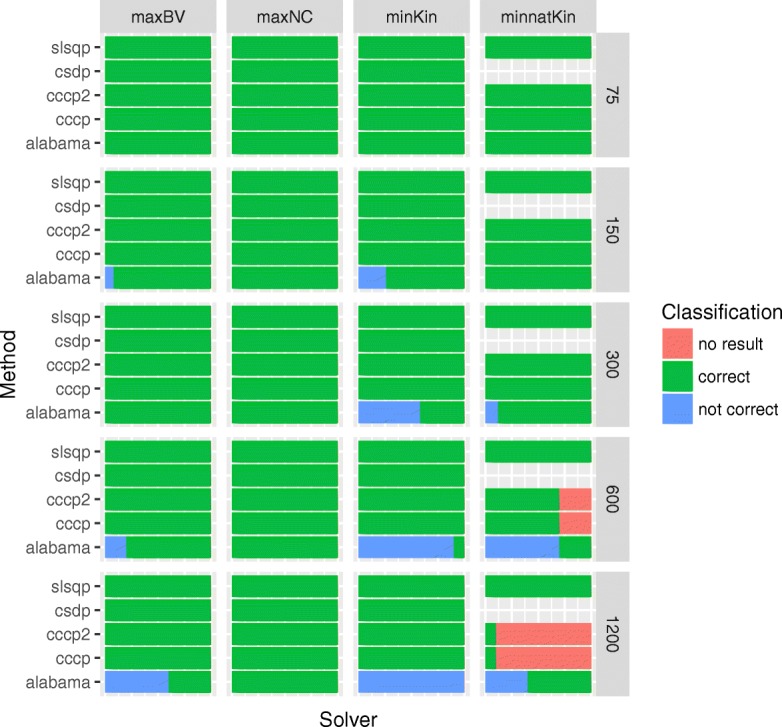
Classification of results. Proportion of correct results (green), the proportions of suboptimal results (blue), and the proportions of cases in which no feasible solution was found (red) for different solvers, OCS-methods and numbers of selection candidates. A solution was classified as not correct if the value of the objective function at the solution deviates from the best estimate by more than 1%

**Fig. 3:**
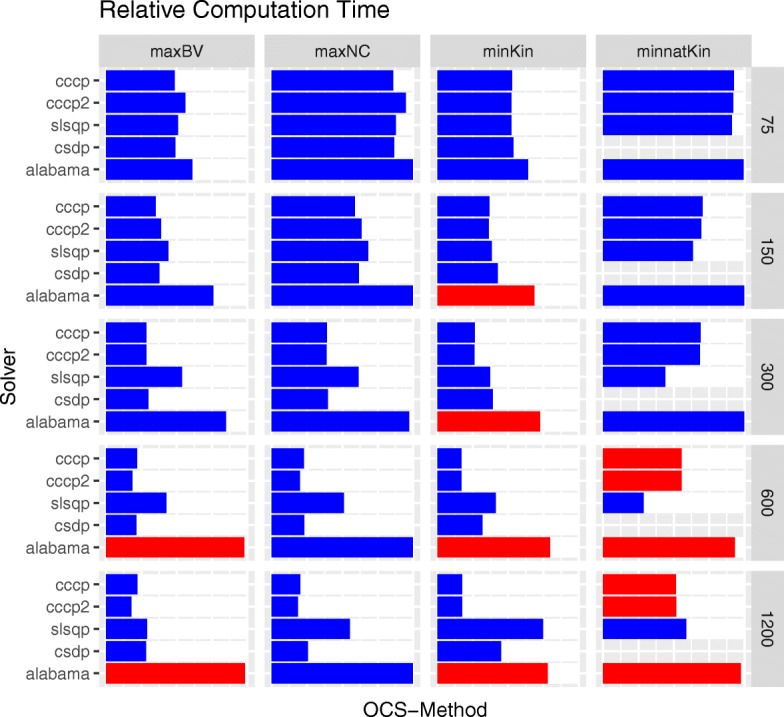
Relative computation time. Relative computation time needed by different solvers to find optimum solutions for different OCS-methods. Computation times are standardized and can be compared directly only for a given number of selection candidates, which are displayed at the right-hand side. Bars representing computation times of solvers that did not produce correct results in at least 80% of the cases are red

The results shown in Figs. 2 - 3 were obtained from 50 replicates for scenarios with less than 300 selection candidates, and from 10 replicates for scenarios with more than 300 selection candidates. Figure 2 shows the proportions of correct results (green), the proportions of suboptimal results (blue), and the proportions of cases in which no feasible solution was found (red). These proportions are shown for the different solvers, OCS-methods, and numbers of selection candidates. A result was classified as correct if the ratio between the value found by the solver and the best solution deviates from one by less than 1%. Figure 3 shows the relative computation times needed by the different solvers. Computation times are standardized and can be compared directly only for a given number of selection candidates. Bars representing computation times of solvers that did not produce correct results in at least 80% of the cases are red.

All solvers were able to find correct solutions when the number of selection candidates was small. Solver alabama provided suboptimal results for larger optimization problems and had the longest runtime, so its use can not be recommended. Solvers cccp and cccp2 had the shortest runtime for problems with linear or quadratic objective function and provided correct results, so their use can be recommended for breeding programs that aim at maximizing genetic gain, at recovering the native genetic background, or at minimizing kinships.

Minimization of the native kinship is in general not a convex problem, so solver csdp could not be used for this. Solvers cccp and cccp2 are also not designed to solve non-convex problems, but were able to find the solution when the number of selection candidates was small. When the number of candidates was large, then their solutions did not satisfy the constraints. Hence, only solver slsqp can be recommended for breeding programs that aim at maximizing the genetic diversity at native alleles.

### Computation of pedigree-based kinships

Different R packages exist to compute pedigree-based kinships, or, equivalently, the additive relationship matrix **A**. Table 1 shows the computation time needed to compute the kinship matrix for different numbers of individuals. The pedigree size was the number of individuals included in the pedigree, which are the individuals for which the kinships were to be computed and their ancestors. R package **optiSel** was 10 times faster than all other packages. Moreover, all other packages failed to compute the kinship matrix for the example data set with 32698 individuals because the memory that would have been needed by those packages was larger than 32 GB RAM.

**Table 1:** Time needed for computing kinship matrices on a 3.40 GHz PC with 32GB RAM

Pedigree size	Individuals	nadiv	optiSel	Pedigree	pedigreeR	pedigreemm
47064	4705	189	13	648	184	184
70075	12269	1082	64	-	930	1080
96411	32698	-	153	-	-	-

## Conclusion

Optimum contribution selection applied to local breeds requires special attention due to the conflicting objectives of their breeding programs. The free R package **optiSel** is an easy-to-use software taking these conflicting objectives into account. It enables to estimate the genetic parameters that need to be controlled, and which can subsequently be used to define the objective and constraints of a breeding program. The optimization problem can be solved with a variety of solvers, which provide a list with the optimum numbers of offspring for all selection candidates, and which can subsequently be used for mate allocation.

## Availability and requirements

Project name: optiSel 2.0.1

Project home page: https://CRAN.R-project.org/package=optiSel

Operating system(s): Platform independent

Programming language: R and C++

Other requirements: None

License: The software is free

## Additional file

Additional file 1 Example data set and replication script. (ZIP 14700 kb)

## References

[1] Meuwissen THE (1997). Maximising the response of selection with a predefined rate of inbreeding. J Animal Sci.

[2] Hartwig S, Wellmann R, Hamann H, Bennewitz J (2014). The contribution of migrant breeds to the genetic gain of beef traits of german vorderwald and hinterwald cattle. J Anim Breeding Genet.

[3] Hartwig S, Wellmann R, Emmerling R, Hamann H, Bennewitz J (2015). Short communication: Importance of introgression for milk traits in the german vorderwald and hinterwald cattle. J Dairy Sci.

[4] Amador C, Toro MA, Fernandez J (2011). Removing exogeneous information using pedigree data. Conserv Genet.

[5] Wellmann R (2012). Optimum contribution selection for conserved populations with historic migration. Genet Sel Evol.

[6] Bennewitz J, Simianer H, Meuwissen THE (2008). Investigations on merging breeds in genetic conservation schemes. J Dairy Sci.

[7] Wang Y, Wellmann R, Bennewitz J. Novel optimum contribution selection methods accounting for conflicting objectives in breeding programs for livestock breeds with historical migration. GSE. 2017; 49:45. 10.1186/s12711-017-0320-7 PMC5427594 28499352

[8] Wang Y, Segelke D, Emmerling R, Bennewitz J, Wellmann R (2017). Long-term impact of optimum contribution selection strategies on local livestock breeds with historical introgression. G3.

[9] Meuwissen THE (2002). GENCONT: An operational tool for controlling inbreeding in selection and conservation schemes. Proc. 7th World Congr. Genet. Applied to Livest. Prod., Montpellier, France.

[10] Pong-Wong R, Woolliams JA (2007). Optimisation of contribution of candidate parents to maximise genetic gain and restricting inbreeding using semidefinite programming. Genet Sel Evol.

[11] Berg P, Nielsen J, Sørensen MK. EVA: Realized and predicted optimal genetic contributions. Proc 8th World Cong Genet Appl Livest Prod Belo Horizonte, Brazil. 2006;:246.

[12] Kinghorn BP (2011). An algorithm for efficient constrained mate selection. Genet Sel Evol.

[13] Fujisawa K, Kojima M, Nakata K, Yamashita M. SDPA (SemiDefinite Programming Algorithm) user’s manual—Version 6.0; 2002. Research Report B-308, Dept. of Mathematical and Computing Sciences, Tokyo Institute of Technology, Oh-Okayama, Meguro, Tokyo 152-8552, Japan, 1995. Revised July 2002.

[14] Varadhan R. Alabama: Constrained Nonlinear Optimization. 2015. R package version 2015.3-1. https://CRAN.R-project.org/package=alabama.

[15] Borchers B (1999). Csdp, a c library for semidefinite programming. Optim Methods Softw.

[16] Kraft D. A software package for sequential quadratic programming. 1988. Tech Rep DFVLR-FB 88-28, DLR German Aerospace Center—Institute for Flight Mechanics, Köln, Germany.

[17] Pfaff B. The R package cccp: Design for solving cone constrained convex programs. R Financ 16-17 May 2014 Chic. 2014.

[18] Maignel L, Boichard D, Verrier E (1996). Genetic variability of french dairy breeds estimated from pedigree information. Interbull Bull.

[19] Peripolli E, Munari DP, Silva MVGB, Lima ALF, Irgang R, Baldi F (2016). Runs of homozygosity: current knowledge and applications in livestock. Anim Genet.

[20] Ferenčaković M, Sölkner J, Curik I. Estimating autozygosity from high-throughput information: effects of snp density and genotyping errors. Genet Sel Evol. 2013;45(42). 10.1186/1297-9686-45-42 PMC4176748 24168655

[21] Meuwissen THE (2009). Genetic management of small populations: A review. Acta Agric Scand Sect A.

[22] Falconer DS (1989). Introduction to Quantitative Genetics.

